# Mobilizing a Kingdom During a Pandemic: The Health Marketing Campaigns Applied by the Saudi Ministry of Health to Promote (COVID-19) Vaccine Confidence and Uptake

**DOI:** 10.7759/cureus.53734

**Published:** 2024-02-06

**Authors:** Najla Jazzaa Alhraiwil, Lamay Alghaith, Walid Alharbi, Sarah AlAjaji, Anas Alhumaid, Mohammed S Aldossary

**Affiliations:** 1 Public Health, Saudi Arabia Ministry of Health, Riyadh, SAU; 2 Communication, Saudi Arabia Ministry of Health, Riyadh, SAU; 3 Research and Studies, Saudi Arabia Ministry of Health, Riyadh, SAU

**Keywords:** ksa:kingdom of saudi arabia, covid-19 vaccination, awareness campaign, covid-19, social marketing

## Abstract

Background: COVID-19 vaccination hesitancy is threatening the global intended herd immunity. Social marketing integration rocketed in promoting public health through awareness campaigns. Saudi Arabia was one of the countries that used social marketing to promote COVID-19 vaccinations for all age groups through successive campaigns. This study aims to describe the content of the campaigns held by the Saudi Ministry of Health to promote COVID-19 vaccine uptake.

Methods: A track’s working strategy was created to contain COVID-19 spread in Saudi Arabia followed by a vaccination track. Six tracks were maintained over six months extended from June 2020 to December 2020. As a result, different campaigns were launched, and key performance indicators were identified and collected. Data from campaigns and key indicators were collected to determine outreach and impact.

Results: Five campaigns were initiated receiving high interactions from governmental entities and the public. The individuals’ percentage who received full vaccination doses and booster vaccinations increased. Moreover, the last campaign promoting vaccinations in children achieved a 60% willingness rate among adults to vaccinate their children.

Conclusion: COVID-19 awareness campaigns achieved successful outcomes in Saudi Arabia and currently the Kingdom sustained higher vaccination proportions than the average vaccination attainment worldwide.

## Introduction

The COVID-19 pandemic triggered health systems worldwide, and response effectiveness differed within and between countries around the globe owing to the available resources and the population at risk [1]. The health and economic costs of the pandemic have been deleterious with marked disparities among the regions of the globe due to different preparedness, prevention, and control strategies [2]. A notation that represented an interesting paradox during the pandemic belonged to Taiwan, given its proximity to China. Taiwan efficiently and effectively controlled the COVID-19 pandemic effects, due to the government's early confinement and preventive strategies and the population's abidance with early preventive measures and strict precautions [3]. Moreover, among the countries that successfully responded to the COVID-19 pandemic was the Kingdom of Saudi Arabia (KSA), the largest country in the Arabian Peninsula [4].

Since its emergence, the COVID-19 pandemic was considered a public health emergency that requires delineated attention and control by concerned bodies for national risk management. In light of this and after the first COVID-19 case encounter in KSA, the governing bodies rushed to face and ameliorate the consequence of the dire situation [5]. Amidst a health system transformation period, KSA rapidly utilized resources and implemented precautionary and preparedness strategies to face the pandemic - protecting both Saudi and non-Saudi individuals [6,7]. This was manifested by equal distribution of health services, mainly by public and non-public institutions, such as free COVID-19 testing and proper treatments [6,8].

KSA Ministry of Health adopted the strategies set by the World Health Organization (WHO) to face the pandemic by utilizing the preparedness and nine response strategic pillars of public health [7], including: Country-level coordination, planning, and monitoring; Risk communication and community engagement; Surveillance, rapid response teams, and case investigation; Points of entry, international travel, and transport; National laboratories; Infection prevention and control; Case management; Operational support and logistics; in addition to the added pillar, Maintaining essential health services and systems [7,9]. Accordingly, Saudi authorities took the responsibility for proper governing of the spread, response, and containment of the COVID-19 virus [7].

KSA has followed all national and international updates relevant to controlling the COVID-19 pandemic. COVID-19 vaccination was at the top of the list on the KSA Ministry of Health agenda for combatting the pandemic. The first two vaccines that were approved for use in the KSA were Pfizer and AstraZeneca in December 2020 and February 2021, respectively [10]. Vaccinations in KSA were free of charge to all KSA citizens and residents. Since disparities in COVID-19 have emerged during the pandemic where minorities were affected the most, KSA annihilated this disproportionality not only by providing equity in healthcare but also in vaccine distribution [10,11].

The acceptance rate of the vaccine ahead of vaccination ranged between 40% and 65% [12-14] and was also shown to be higher among healthcare workers, where more than 70% of healthcare workers expressed their acceptance [15]. Social media platforms were also used to assess the people’s willingness to get vaccinated and to dissolve some complicated thoughts and misinformation propagating about COVID-19 vaccination and its effectiveness through legit posts by the Ministry of Health and other health authorities [5,16].

The use of social media in health promotion and health campaigns has been used early on [17,18]. COVID-19 vaccination campaigns have paved their way through this communication tool all over the world and became one of the focuses of healthcare professionals and healthcare facilities’ administrators [19]. KSA is one of the countries that utilized several social media platforms, mainly Twitter as the cornerstone, to reach out to the highest number possible of its population [16,20]. Despite that fake and low-quality health information is popular on social media platforms [21], the KSA Ministry of Health maintained its legitimacy by publishing through trustworthy accounts and spreading public visibility. The Ministry of Health also made available an online electronic system for automated vaccine appointments through “Sehhaty” Application which is the Arabic translation for “My Health” [10].

A total of five vaccination campaigns were conducted in KSA so far. The first and the second aimed toward achieving complete vaccination for all eligible individuals. The third called for the booster dose, and the fourth and the fifth fostered the booster dose in the elderly and called for the vaccination in children, respectively. KSA has established so far 68,148,406 vaccine doses out of which 77.4% received at least the first dose [22,23]. In this report, we will present insights into these vaccination campaigns, their aims, and outputs, as well as their impact on vaccination acceptance and uptake in KSA.

## Materials and methods

In the war room

**Figure 1:**
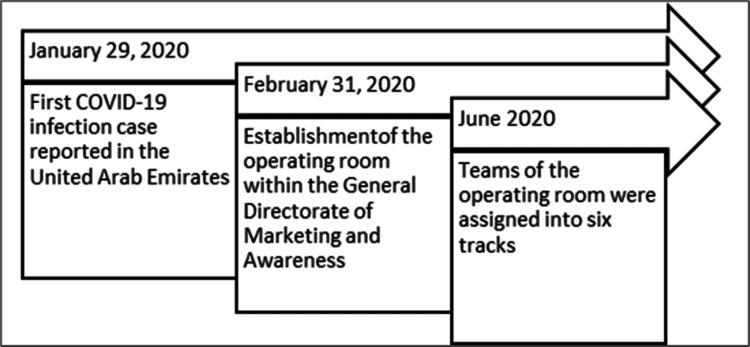
Timeline toward creating the Track’s Working Strategy

An operating room was established within the General Directorate of Marketing and Awareness (recently empowered to become the Assistant Deputyship for Communication) just after the first confirmed COVID-19 infection in the Middle East region and one month before the first confirmed case in the country. Thereafter, the efforts of the communication team have shifted toward the COVID-19 pandemic shortly after the confirmation of the first case in KSA. The operating room consisted of 20 experts who represent the internal departments and coming with different specializations. For instance, marketing, media, public health, health education, and project management specialists. In June 2020, an evaluation process was initiated. The initial evaluation of pre-assigned teams’ performance reflected discrepancies in the level of efficiency between internal departments. Consequently, ending up assigning teams to six tracks that were created based on the targeted audience (Figure 1).

**Figure 2:**
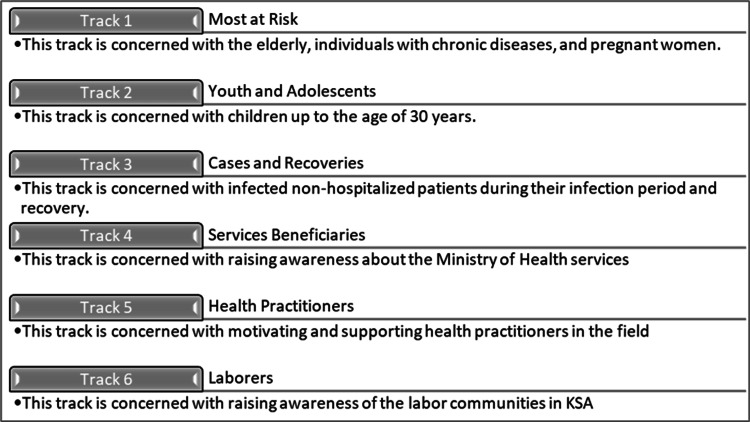
The Six Tracks created by the General Directorate of Marketing and Awareness.

The tracks are summarized in “Figure 2”. Each track had at least four team members with different specializations and years of experience. These tracks were supported with administrative services by two teams, the Quality and Publication Team and the Measurement Team.

In addition, four key performance indicators were evaluated biweekly by the project management office to ensure the effectiveness of the teams and evaluate their performance. The activities of these six tracks were maintained over six months extended from June 2020 to December 2020, and the mission of the tracks was considered phase one. In January 2021, the teams were honored for their efforts and shifted back to their regular state “business as usual” and the tracks’ number was minimized to the Awareness Department Team and the Marketing Department Team.

In parallel, and after the emergence of the COVID-19 vaccine, a Vaccination Track was established. It consisted of 10-11 members from both department teams. In June 2021, along with the start of the second COVID-19 pandemic wave, the Precautions Track was established to promote commitment to preventive measures. This track lasted for four months then switched into an operational project. However, the mission of the Vaccination Track continued until the first quarter of the year 2022.

## Results

Description of the campaigns

**Figure 3:**
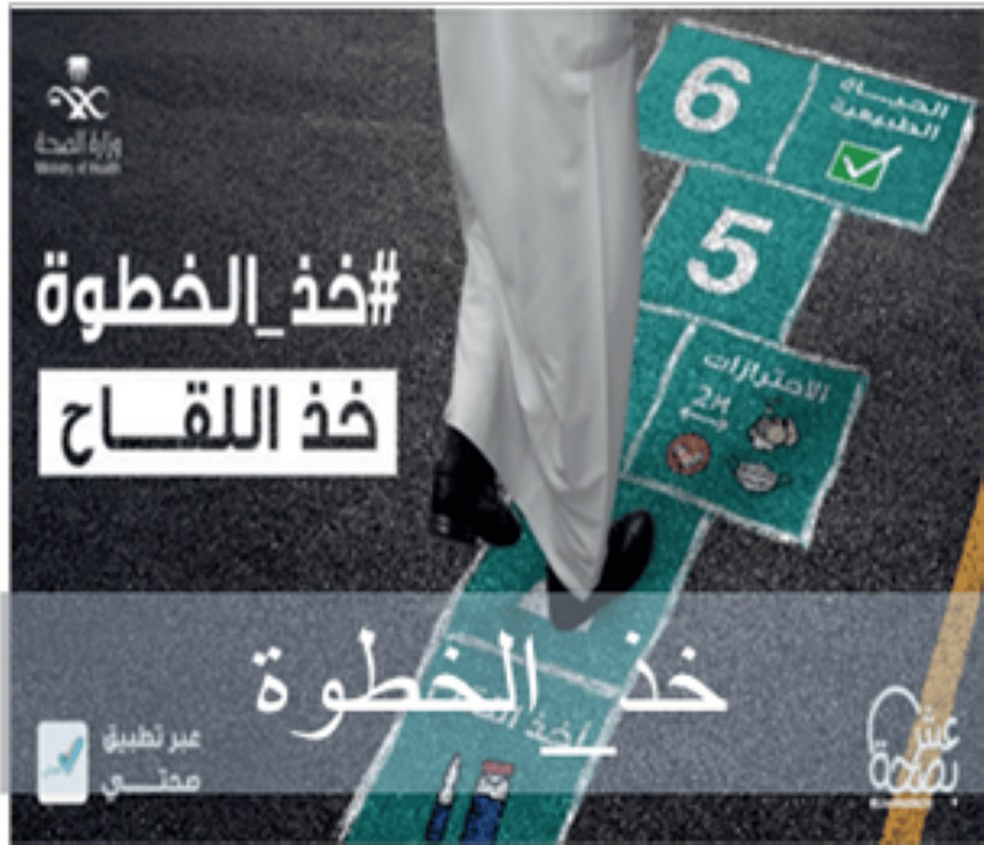
Campaigns' slogans in the original language "The Arabic Language" Title : "Take the Step" used in the first campaign. #take the step take the vaccine

**Figure 4:**
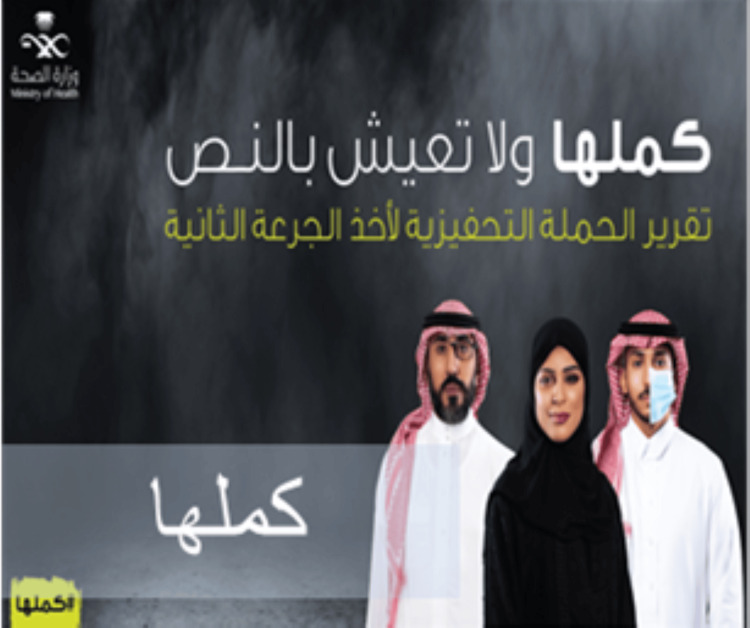
Campaigns' slogans in the original language "The Arabic Language" Title: "Complete It" used in the second campaign. Complete it and don't live in the middle The awareness campaign to promote the second vaccination dose.

**Figure 5:**
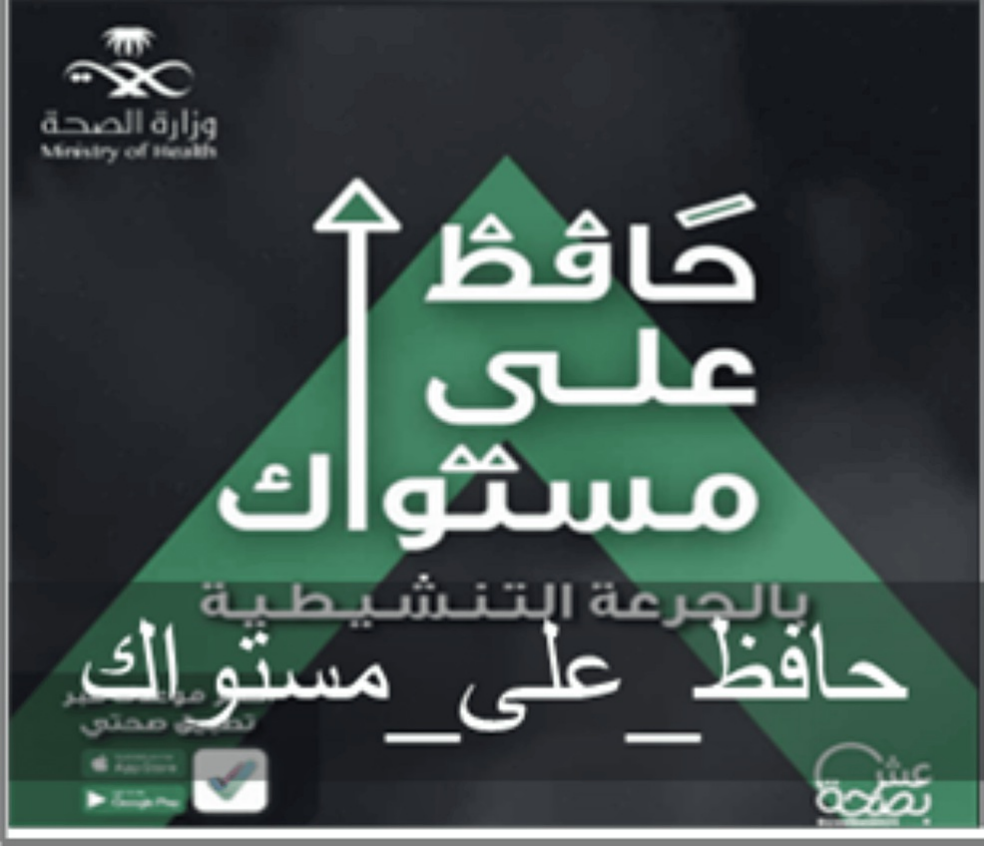
Campaigns' slogans in the original language "The Arabic Language" Title: "Maintain your levels"  used in the third campaign. Maintain your levels with the Stimulant dose

**Figure 6:**
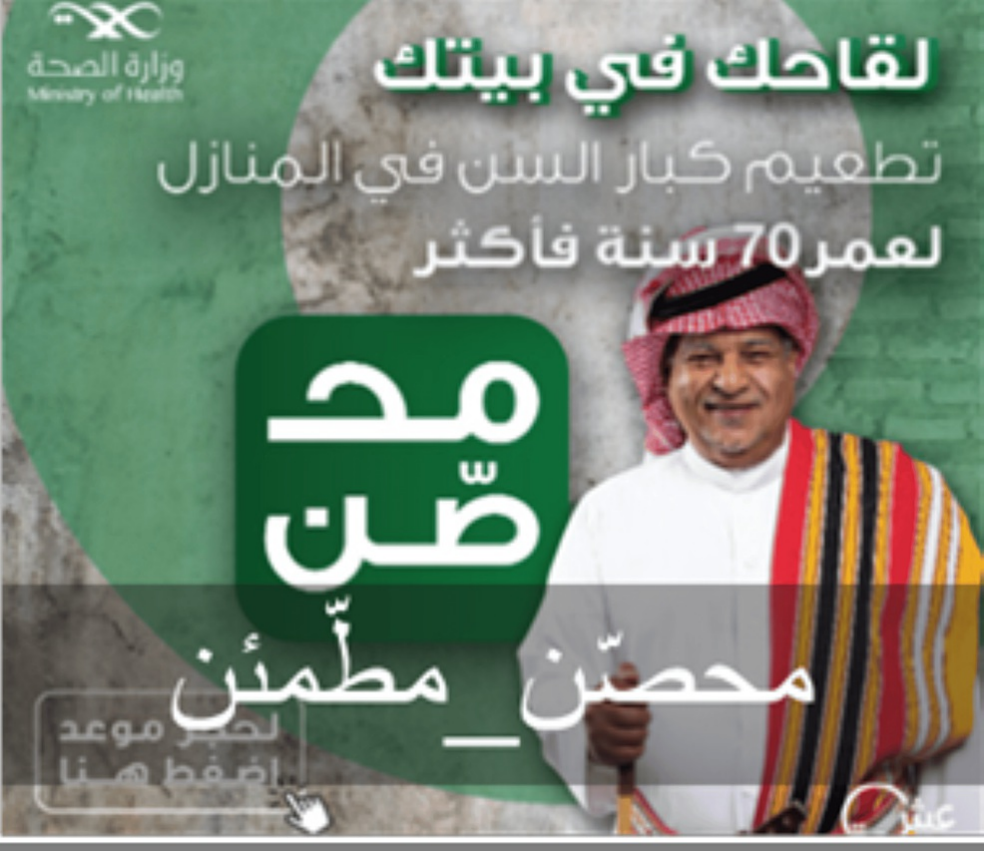
Campaigns' slogans in the original language "The Arabic Language" Title: "Immune and reassured” used in the fourth campaign. Your vaccine at your home Vaccinating the elderly at home

**Figure 7:**
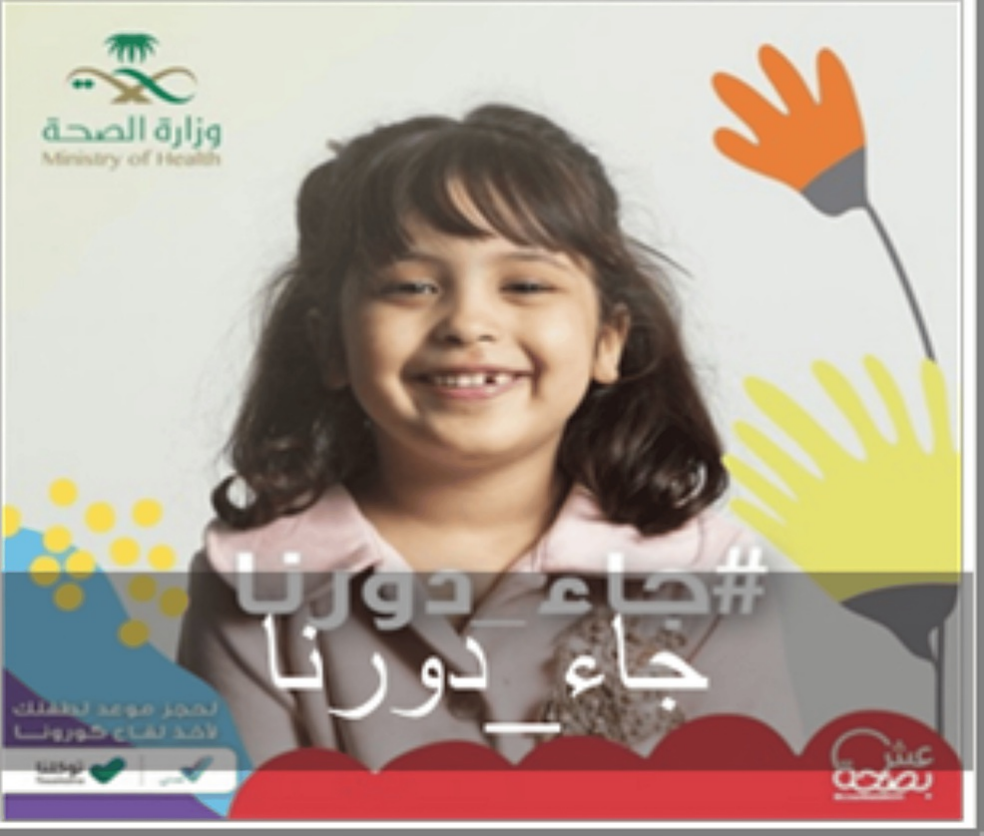
Campaigns' slogans in the original language "The Arabic Language" Title “It is our turn now” used in the fifth campaign.

Five main COVID-19 vaccination campaigns were conducted in KSA between March 2021 and January 2022. Figures 3-7 present an overview of the campaigns’ slogans in the original Arabic language. The first campaign held the title “Take the step”, the second “Complete it,” the third “Maintain your levels,” the fourth “Immune and reassured,” and the fifth “It is our turn now.” The common aim of all the campaigns was to establish herd immunity and protect from COVID-19 to the maximum extent possible. All the campaigns were accompanied by a hype-up teaser video as the main video of the campaign and exceptionally, the fifth campaign had two main videos. In addition, the campaign’s hashtags utilized most of the useful features social media platforms offer accompanied by spot storytelling videos appealing to the public.

*Audio-Visual Reach* 

All the campaigns were accompanied by combinations of short voice messages and mobile texts as well as TV and radio ads, outdoor street advertisements, billboards, and placards distributed across the different regions of the kingdom. In addition to tremendous posts on Twitter, the campaigns also made use of other social media platforms among which was Snapchat. Similar to the first campaign publicization strategies through social media platforms, short videos, outdoor billboards, and posters as well as TV and radio advertisements. Another interesting involvement was by the private sector like coffee shops, cinemas, and banks, whereby they also publicized for the campaigns by posters and advertisements.

The main video of the first campaign utilized the famous “hopscotch” game that starts with a cell written on it “first vaccination” and ends with two cells marked with “normal life” and “herd immunity” phrases. The “hopscotch” paradigm was also posted in different places on the floors of malls, cafeterias, and other public places. Twelve restaurants and coffees integrated the campaign slogan and paradigm design into a designated campaign barcode. The campaign also created competitions for children using the “hopscotch” game. The Ministry of Health also collaborated with the Ministry of Commerce by providing discounts on services from more than 290 companies for those who took the COVID-19 vaccine.

Unique Launch of the Second Campaign

The launching of the second campaign was unprecedented. The Ministry of Health and the Ministry of Sports created a surprising breakthrough during the entrance of two football teams (Al-Nasr and Al-Etihad) with only half the players into the field. This drew the attention of the audience everywhere in the kingdom to the importance of completing their vaccination profile by taking the second dose. They supported the performance with the hashtag “don’t settle halfway, complete it.”

Booster Campaigns

The third campaign was conducted to raise awareness about the first booster dose in an attempt to reach herd immunity after the first two doses’ uptake. They also designed leaflets with awareness-raising quotes and schemes and various medical information related to the importance of the booster dose against newly emerging COVID-19 variants.

The fourth campaign was an additional campaign to raise awareness about the vaccine’s second booster uptake. This campaign also broadcasted a main video that was dedicated to vaccination in the elderly. The video was very friendly with a great sense of humor making it very appealing to the public. In addition to the main video, the campaign also created main showcase posters of two elderlies, a man and a woman advocating for the vaccine reuptake.

Children Vaccination Campaign

The fifth campaign activities were tailored to children aged five to 11 years. The targets of this campaign included achieving a high percentage of vaccine uptake by children, spreading, and delivering knowledge about the service, and encouraging parents for vaccinating their children. Eye-catching campaign-related posts of children were published on Twitter along with other posts that included calls for action to get vaccinated through registration on “Sehhaty” and “Tawakkalna” applications. “Sehhaty” platform provided by the Ministry of Health constitutes a hub for a variety of health services that include - Healthcare steps, Easy access to health services, Healthy family services, Health lifestyle services, and Prevention/protection services. The latter has substantial use and utilization during the COVID-19 pandemic where it facilitated COVID-19 screening and vaccination appointments [24]. “Tawakkalna” was created by KSA Saudi Data and Artificial Intelligence for multiple uses during the COVID-19 pandemic in an attempt to reduce the pandemic spread in KSA; interestingly, the “Caution Mode” built within the application allows users detect a close by infected/suspected/exposed user [25]. As a support to this campaign, the Saudi Press Agency announced on January 16, 2022 through their news page, the availability of the COVID-19 vaccine to children from five to 11 years old. The YouTube channel “Livewellmoh” and The Saudi Ministry of Health on Twitter shared the campaign’s main video which is very tempting to children. As an illustration, the video's subject matter is that children getting vaccinated transform into heroes after vaccination.

Outreach and impact

**Figure 8:**
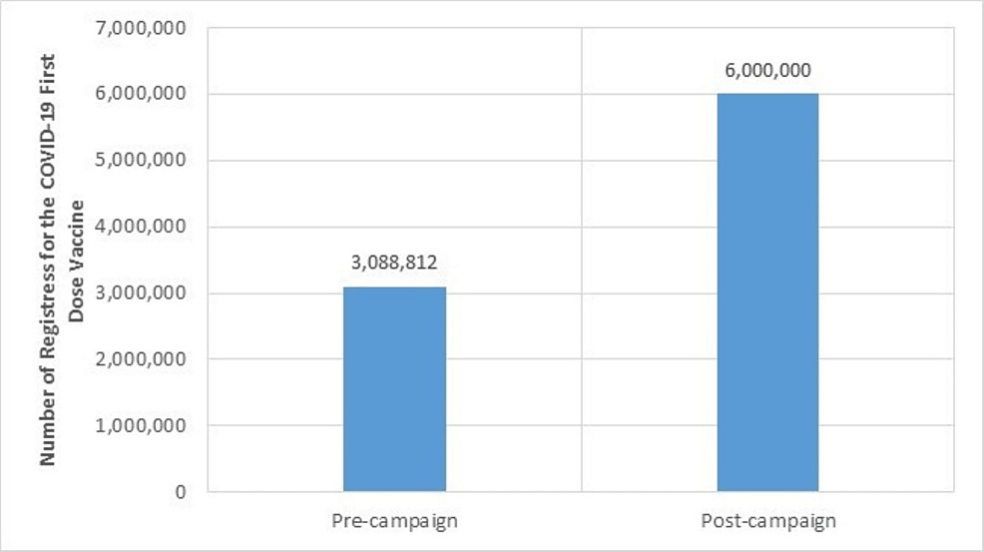
Effect of the COVID-19 first vaccination dose campaign on registration numbers

The inaugural campaign received involvement from more than 30 governmental entities, had more than 14.5 million video views, more than 5.6 million interactions with the campaign posts, and nine million views of the campaign leaflets as well as extensive postings by recognized Saudi sports leagues. In addition, the campaign-initiated communications through 13 sites and applications reached more than 18.7 million people. As a result, an increase in vaccine uptake was documented after the introduction of this first campaign. Particularly, pre-campaign vaccine registrations of 3,088,812 increased after the campaign up to 6,000,000 within less than a month (Figure 8).

**Figure 9:**
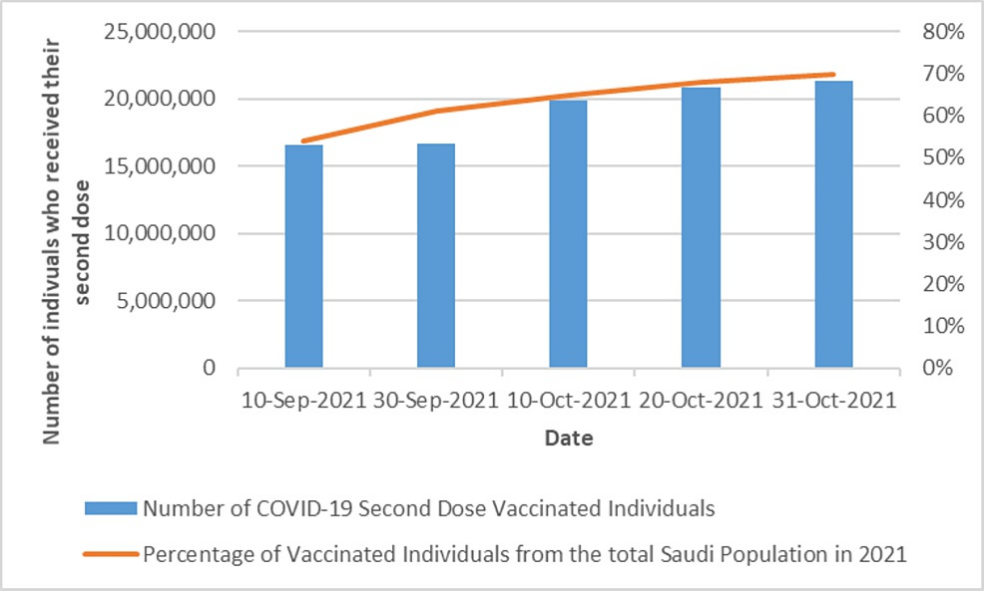
Effect of the second COVID-19 vaccination campaign on vaccination numbers and percentages

Additionally, the second campaign also received similar interactions and reached more than 30 million appearances and 11.4 million views by the public. Subsequently, the second campaign increased the percentage of fully vaccinated individuals to more than 70% after the campaign execution (Figure 9).

**Figure 10:**
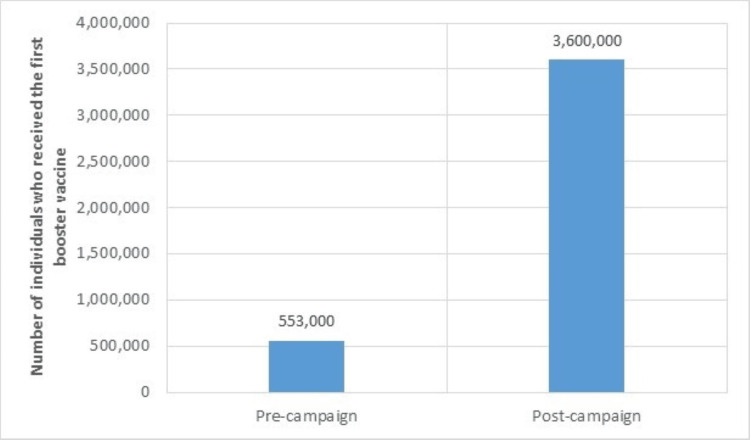
Effect of the COVID-19 third vaccination (booster) campaign on vaccination numbers

Moreover, the booster dose that was advocated for by the third campaign that reached more than 5.5 million views, was taken by more than additional 3.6 million people (Figure 10).

**Figure 11:**
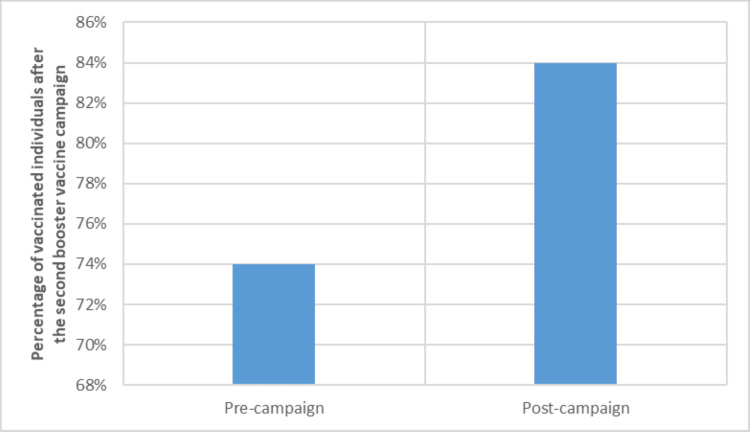
Effect of the fourth vaccination (second booster) campaign on vaccination percentages

The fourth campaign achieved a 10% increase in the number of individuals who received the second booster (Figure 11). The last in this series was the fifth campaign that specifically targeted the pediatric population. In total, the fifth campaign reached the public through more than six million appearances and received more than 500 thousand interactions. The community showed diverse ways of responding and interacting with the campaign mostly prominent through Twitter.

To dig more deeply into the impact of this campaign, The Ministry of Health and the National Center for Public Opinion Polls initiated a survey during November 2021 to collect data about the community’s opinions of this campaign. The sample for this survey was chosen using cluster randomization from around the different regions of the kingdom and targeted individuals who are 20 years and older of both genders.

**Figure 12:**
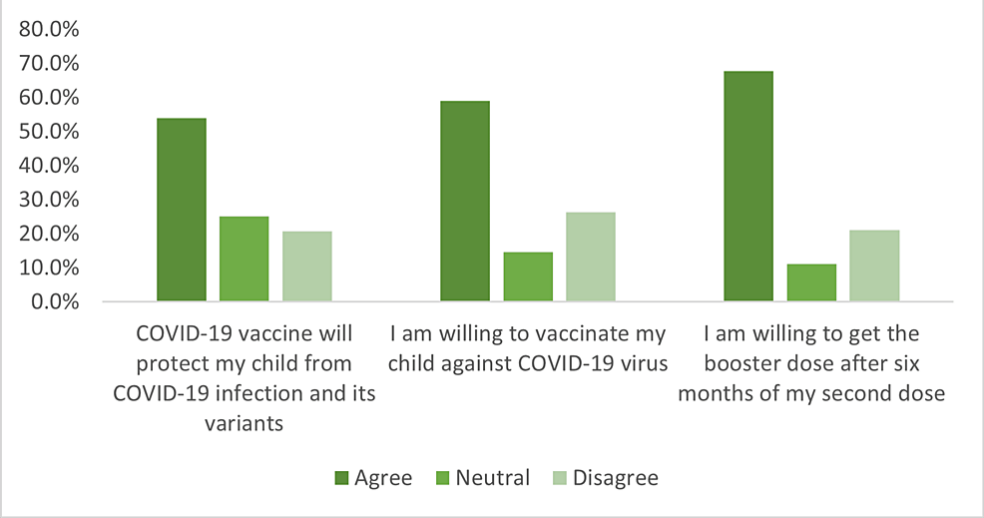
The results of the COVID-19 vaccination-related questions from the fifth COVID-19 vaccination awareness campaign survey

A total of 1,105 individuals participated and had their data analyzed (Table 1); the majority were males (77%) and all participants have had children within the five to eleven years old range. Fifty-four percent of the participants agreed with the phrase “The COVID-19 vaccine will protect my child from COVID-19 viral infection and its variants” and 59% showed interest in vaccinating their children. Sixty-eight percent showed willingness toward getting a third dose of the vaccine six months after the second dose and 70% considered themselves abiding by the facemask usage in indoor spaces with the proper use of hand hygiene (Figure 12).

**Table 1:** Baseline characteristics of the participants in the fifth COVID-19 vaccination awareness campaign survey.

Characteristics		N=1105
Gender	Male	848 (76.6%)
	Female	259 (23.4%)
Marital Status	Married	1023 (92.6%)
	Single	45 (4.1%)
	Divorced	31 (2.8%)
	Widowed	6 (0.5%)
Age Category	≤30 years	75 (6.8%)
	31-40 years	384 (34.8%)
	41-50 years	477 (43.2)
	≥50 years	167 (15.1)

## Discussion

This is the first report on the different vaccination campaigns held in the Kingdom of Saudi Arabia. In this report, we analyzed the impact of COVID-19 vaccination awareness campaigns held in KSA. The campaigns were highly beneficial, and the Ministry of Health successfully spread awareness among KSA citizens and achieved vaccination coverage for roughly 70% of the Saudi population with at least two doses. According to the WHO, hesitancy toward vaccination is listed among the top global health threats [26]. The strategies followed by the Saudi Ministry of Health to raise awareness and promote acceptance of the COVID-19 vaccination were goal-oriented within the social marketing context of promoting health protection actions. Social media influence on COVID-19 pandemic health behaviors has been demonstrated to be effective [27]. Social media platforms played an important role in the dissemination of the campaigns’ goals and messages, particularly Twitter. This is in line with other studies whereby Twitter has been the most used and main platform for collecting COVID-19 data and perceptions of vaccination [19,28]. A recent qualitative content analysis study was conducted to examine Twitter’s use and impact during the COVID-19 pandemic in the KSA [16]. The study examined two groups of tweets, disease-related and non-disease-related topics, and highlighted the extensive efforts made by the government ministries. In the current analysis of the campaigns, we reported on the activities and several collaborations the Ministry of Health performed. These include spreading hashtags and mainstream videos related to the different campaigns which were very useful to the users. A cross-sectional study was conducted in Makkah from February 23 to March 2, 2021, which is the period during the first COVID-19 vaccination awareness campaign [29]. It showed that the citizens were not aware of COVID-19 vaccination importance and effectiveness as much as they were knowledgeable of COVID-19 infection, symptoms, and prevention strategies. Less than 40% of the studied sample showed interest and willingness in receiving the COVID-19 vaccine. Interestingly, after the first awareness campaigns, recognized increases in vaccine registration and uptake by Saudi citizens were observed. For instance, an increase of 70% in registration after the first campaign and a full vaccination population coverage of more than 70% after the second campaign. This coverage is higher than the average COVID-19 full vaccinations worldwide which is 65.2% [30]. This communication strategy was able to reach a large number of people thus creating a link between the public and policies as well that were updated regularly [16,31]. Empowerment and health behavior were the most prominent and received the most interactions from the public [16]. The fifth campaign, which was conducted exclusively for children, reached two million views on Twitter and received more than 1.8 thousand reactions.

Our study results also support the integral role of social media in decreasing the hesitancy toward vaccination. This was demonstrated by the higher percentage (>70%) of those who got fully vaccinated compared to pre-campaign acceptance desires (as low as 40%). This is in line with current findings in the literature that illustrates the power of properly using social media for the sake of addressing COVID-19 vaccination hesitancy [32].

## Conclusions

This study describes the COVID-19 vaccine campaigns and the potential response among the community. However, this does not provide insights on the uptake of the vaccine as a response to campaigns. The community response to vaccines is a combination of several actions and policies implemented by the Saudi ministry.

Therefore, more timely studies should be conducted across different regions of the Kingdom assessing the different factors that might affect the uptake of the vaccine and assess the reachability of the COVID-19 vaccine awareness campaigns and the best ways to be delivered according to the region’s most utilized platforms and/or areas. Attempts to reach higher vaccination rates should be planned for through the digitalized platforms our world is witnessing today. KSA has shown tremendous efforts and achievements in utilizing artificial intelligence technology in an attempt to decrease COVID-19 cases. Attempts were successful and impactful in reducing the pandemic's spread, especially among young adults.
